# Activation and Inhibition of Human Matrix Metalloproteinase-9 (MMP9) by HOCl, Myeloperoxidase and Chloramines

**DOI:** 10.3390/antiox11081616

**Published:** 2022-08-20

**Authors:** Yihe Wang, Christine Y. Chuang, Clare L. Hawkins, Michael J. Davies

**Affiliations:** Department of Biomedical Sciences, Panum Institute, University of Copenhagen, 2200 Copenhagen, Denmark

**Keywords:** matrix metalloproteinase (MMP), extracellular matrix, myeloperoxidase, hypochlorous acid, chloramines, tissue inhibitor of matrix metalloproteinase (TIMP), gelatinase, protein oxidation, matrix turnover, proteolysis

## Abstract

Matrix metalloproteinase-9 (MMP9, gelatinase B) plays a key role in the degradation of extracellular-matrix (ECM) proteins in both normal physiology and multiple pathologies, including those linked with inflammation. MMP9 is excreted as an inactive proform (proMMP9) by multiple cells, and particularly neutrophils. The proenzyme undergoes subsequent processing to active forms, either enzymatically (e.g., via plasmin and stromelysin-1/MMP3), or via the oxidation of a cysteine residue in the prodomain (the “cysteine-switch”). Activated leukocytes, including neutrophils, generate O_2_^−^ and H_2_O_2_ and release myeloperoxidase (MPO), which catalyzes hypochlorous acid (HOCl) formation. Here, we examine the reactivity of HOCl and a range of low-molecular-mass and protein chloramines with the pro- and activated forms of MMP9. HOCl and an enzymatic MPO/H_2_O_2_/Cl^−^ system were able to generate active MMP9, as determined by fluorescence-activity assays and gel zymography. The inactivation of active MMP9 also occurred at high HOCl concentrations. Low (nM—low μM) concentrations of chloramines formed by the reaction of HOCl with amino acids (taurine, lysine, histidine), serum albumin, ECM proteins (laminin and fibronectin) and basement membrane extracts (but not HEPES chloramines) also activate proMMP9. This activation is diminished by the competitive HOCl-reactive species, methionine. These data indicate that HOCl-mediated oxidation and MMP-mediated ECM degradation are synergistic and interdependent. As previous studies have shown that modified ECM proteins can also stimulate the cellular expression of MMP proteins, these processes may contribute to a vicious cycle of increasing ECM degradation during disease development.

## 1. Introduction

Acute and chronic inflammation are associated with multiple human pathologies. Sites of inflammation are characterized by the accumulation of leukocytes, including neutrophils, monocytes and macrophages. The activation of these cells by pathogens, cell signals or numerous other factors, including damaged biomolecules, results in the generation of superoxide radicals (O_2_^•−^) via a “respiratory burst” that is mediated by NADPH oxidase enzymes [1]. These radicals subsequently undergo rapid dismutation to produce hydrogen peroxide (H_2_O_2_). Concomitantly, the heme enzyme myeloperoxidase (MPO) is released into phagolysosomes and also extracellularly [2]. This enzyme has a high surface positive charge and binds avidly to negatively charged macromolecules, including plasma proteins (e.g., caeruloplasmin, low-density lipoprotein) and proteins, glycoproteins, proteoglycans and glycosaminoglycans of the extracellular matrix (ECM) [3]. This results in an accumulation of MPO in ECM structures [3,4,5].

MPO employs H_2_O_2_ and halide or pseudohalide anions to generate potent reactive oxidants, such as hypochlorous acid (HOCl) in the case of chloride ions (reviewed [6]). HOCl, and other members of the hypohalous acid family (e.g., HOBr and HOSCN, formed from Br^−^ and SCN^−^, respectively), can modify and damage both the surrounding cells and the components of the ECM [3,6]. Damage to the ECM is postulated to be particularly severe as a result of the binding of MPO to these materials and the high reactivity of HOCl [7], which limits its diffusion from the site of formation, resulting in “site-specific” damage [3,5]. Amine groups (e.g., the side chains of histidine (His), lysine (Lys) and the N-terminal amine of proteins) are major targets for HOCl due to the reactivity of these side chains [8], their high abundance and the low levels of more reactive targets (e.g., cysteine (Cys), methionine (Met) and selenocysteine) in many ECM and extracellular proteins. Thus, we and others have shown that elevated levels of HOCl-induced modifications can be detected on ECM proteins, glycoproteins and proteoglycans, such as within the artery wall at sites of atherosclerotic lesions [9,10,11,12,13].

The exposure of cells, including naïve (nonoxidant exposed) cells, to modified ECM in vitro alters the gene and protein expression, with the upregulation of the expression of both ECM proteins and matrix metalloproteinases (MMPs) [5,14]. MMP expression is also known to be upregulated in response to a wide range of other biological signals present, or generated by cells, at sites of inflammation [15,16]. These ECM changes modulate the cell adhesion, proliferation, metabolic activity and phenotype [15], with, for example, vascular smooth muscle cells showing evidence for a switch from a quiescent and contractile phenotype to a synthetic and proliferative form [5,14].

MMPs are a large family of enzymes of zinc- and calcium-dependent endopeptidases that degrade ECM proteins [17,18]. Most MMPs are secreted from cells in a latent proMMP form and require activation, with this occurring extracellularly. Conversion to the active form is typically carefully controlled and tightly regulated due to the capacity of these enzymes to generate widespread tissue damage. In general, activation involves a range of proteases, with this being dependent on the specific MMP [17,18]. In the case of MMP9, activation is mediated by proteases including furins, stromelysin-1/MMP3, some cathepsins and plasmin [18,19], which cleave off the inhibitory propeptide. This exposes the active-site Zn^2+^ atom, which is coordinated to the mature protein via three conserved His residues. Cys^7^ in the propeptide sequence acts as a fourth ligand to the His, resulting in the folding of the propeptide sequence over the catalytic site of the enzyme, and preventing enzymatic activity. The activity of MMPs is regulated by tissue inhibitors of matrix metalloproteinases (TIMPs 1–3), with the upregulation of these species often occurring in synchrony with MMP release and activation [20,21].

The activation of some MMPs, and particularly MMP2 and MMP7, has been shown to occur via an alternative mechanism, which involves the oxidation of the conserved propeptide Cys (the “cysteine switch”) [22,23,24]. In particular, it has been shown that HOCl can activate proMMP7, that (unspecified) radicals generated by xanthine/xanthine oxidase can activate proMMP2 [24] and that activated human neutrophils can both activate and inactivate collagenase and gelatinase [25,26,27]. The activation of these proenzymes by oxidation is believed to arise from a loss of the ligating capacity of the cysteine-switch Cys residue to the zinc atom as a result of oxidation, and the release of the constrained propeptide to give an active intermediate form of the enzyme with the propeptide still attached. This oxidation can be either reversible or irreversible, depending on the extent of oxidation [23]. This initial active intermediate form can then be further processed (e.g., by proteolysis) to give the mature form. These data indicate that oxidation may be critical to both the synthesis and excretion of new MMPs (via signaling from damaged ECM) and the activation of latent or newly synthesized MMPs. Oxidative damage and MMP release and activation may therefore be interdependent and result in synergistic damage to the ECMs of tissues.

Considerable evidence has been presented for a key role of MMP9 in ECM damage in multiple pathologies [28,29], including: rheumatoid arthritis [30]; some cancers (where MMP9 plays a role in angiogenesis, invasion, tissue remodeling and metastasis) [31,32]; vascular diseases, including brain ischemia [33]; cardiovascular syndromes, including atrial fibrillation [34], aortic aneurysms [35] and cardiovascular diseases including atherosclerosis [20,36,37,38]. The knockout or knockdown of MMP9, or its inhibition, affords protection against many of these pathologies [39,40].

The study reported here therefore examined whether reagent or MPO-derived HOCl and secondary chloramines formed on amino acids, plasma and ECM proteins would convert recombinant human proMMP9 to active forms of the enzyme. Furthermore, we postulated that preactivated MMP9 might be inactivated by high concentrations of HOCl, as this oxidant reacts rapidly with His residues [8], potentially disrupting the active site of MMP9.

## 2. Materials and Methods

### 2.1. Materials

All chemicals, including *p*-aminophenylmercuric acetate (APMA), NaCl, HEPES, CaCl_2_, Tris, Brij-35, dimethylsulfoxide (DMSO), l-methionine (Met), human serum albumin (HSA), 5,5′-dithiobis (2-nitrobenzoic acid), boric acid and sodium tetraborate decahydrate were from Sigma-Aldrich (St Louis, MO, USA) unless stated otherwise, and all solutions were prepared with Milli-Q grade water (Millipore Advantage A10; Merck-Millipore, Billerica, MA, USA). Myeloperoxidase from human polymorphonuclear leukocytes was purchased from Planta Natural Products (Vienna, Austria). Recombinant human proMMP9 (proMMP9), the profluorescent MMP substrate MCA-Pro-Leu-Gly-Leu-DPA-Ala-Arg-NH_2_ and murine basement membrane extract (BME) were purchased from R&D systems. Novex 10% Zymogram Plus (Gelatin) protein gels (1.0 mm, 10-well), Novex Tris-Glycine SDS sample buffer (2×), Novex Tris-Glycine SDS running buffer (10×), Novex Zymogram renaturing buffer (10×), Novex Zymogram developing buffer (10×) and SimplyBlue SafeStain were purchased from Thermo Fisher. Precision Plus Protein Kaleidoscope prestained protein standard was from BioRad (10–250 kDa). *N*α-acetyl-Lys-OH and *N*α-acetyl-His-NHMe (*N*α-acetyl-His with a *N*-methylamide-derivatized carboxyl group) were purchased from Bachem. Vivaspin concentrators (molecular-mass cutoff: 10 and 50 kDa; Sartorius, Göttingen, Germany) were used for buffer exchange for proMMP9, preactivated MMP9, laminin111 and murine BME.

### 2.2. Detection of MMP9 Activity via Fluorescence Assay

Recombinant human proMMP9 or preactivated MMP9 (both 13 nM) was mixed with increasing concentrations of HOCl (0, 0.5 µM, 1 µM, 2 µM, 5 µM, 10 µM, 50 µM, 100 µM or 250 µM, corresponding to molar excesses of HOCl over proMMP9 of 0, 35, 77, 150, 385, 770, 3850, 7700 and 19200, respectively) in Assay Buffer A (150 mM NaCl, 10 mM HEPES (pH 7.4), 5 mM CaCl_2_) or borate buffer (150 mM NaCl, 5 mM CaCl_2_, 19.4 mM H_3_BO_3_ and 0.14 mM Na_2_B_4_O_7_ · 10H_2_O; pH 7.8) for 24 h at 37 °C. Treatment of proMMP (13 nM) with APMA (1 mM) in Assay Buffer A or borate buffer for 2 h at 37 °C was used as a positive control for MMP9 activation. After activation, the MMP9 samples were diluted 2.5 times with Assay Buffer C (50 mM Tris, 10 mM CaCl_2_, 150 mM NaCl and 0.05% (*w/v*) Brij-35; pH 7.5). A total of 50 µL of the reaction mixture (0.4 ng µL^−1^ proMMP9) was then pipetted into 96-well plates, and the reaction was initiated by adding 50 µL of the profluorescent MMP9 substrate (20 µM in Assay Buffer C). Each well therefore contained 0.26 pmol MMP9 and 10 µM substrate. Substrate and enzyme blank wells were also prepared and analyzed. The fluorescence from the cleaved substrate, expressed as relative fluorescence units (RFUs), was determined using λ_ex_ 320 nm and λ_em_ 405 nm, respectively, in a kinetic mode at 5 min intervals for 1.5 h.

### 2.3. Zymography to Determine Gelatinase Activity of MMP9

ProMMP9 (52 nM) was incubated with increasing concentrations of HOCl, as described above. Subsequently, equal volumes of MMP9 sample and Tris-Glycine SDS Sample Buffer (2×) were mixed, 10 μL (20 ng proMMP9) or 15 μL (6.4 ng preactivated MMP9) of the samples were loaded into each lane on the gel, and the chamber was filled with 1× Tris-Glycine SDS running buffer. After gel electrophoresis at 125 V for 140 min, the gels were incubated with renaturing buffer for 30 min at 21 °C with gentle agitation and were removed. This was then followed by the addition of two successive portions of 1× Zymogram developing buffer, 30 min for the first portion, and then overnight at 21 °C with gentle agitation. The gels were then stained with SimplyBlue Safestain for 1 h at 21 °C, and de-stained using 25% ethanol/10% acetic acid in water for ~30 min until areas of protease activity appeared as white bands against the blue background. The gels were then imaged on a scanner and the band densities were analyzed using ImageJ software (version 1.53s, NIH, Bethesda, MD, USA). Efforts were made to minimize the “maxing out” of the signal of the white bands (i.e., overexposure), although this was difficult to achieve reproducibly. Consequently, the image-analysis data are likely to have greater inherent errors than the fluorescence-activity data (Section 2.2).

### 2.4. Formation and Detection of Chloramines

Amino acid, HEPES and protein chloramines were prepared and kept at 4 °C to minimize decomposition, essentially as described previously [41]. Lys- (Lys CA), HEPES (HEPES-CA) and taurine-chloramines (Tau CA) were prepared by adding HOCl to a 5-fold excess of *Nα*-acetyl-Lys, HEPES and taurine, respectively, in 5 mM sodium phosphate buffer, with a pH of 7.4. Histidine-chloramines (His CA) were prepared by adding HOCl to a 2-fold excess of *N*α-acetyl-His-NHMe. HSA-chloramines, human plasma fibronectin-chloramines (FN CA), laminin111-chloramines (laminin CA) and murine BME-chloramines (bme CA) were generated by adding a 50-fold molar excess of HOCl over the protein concentration. Chloramine concentrations were determined by quantifying the oxidation of 5-thio-2-nitrobenzoic acid (TNB) to 5,5′-dithiobis-(2-nitrobenzoic acid) (DTNB), as described previously [41]. TNB was generated from DTNB by the treatment of 1.3 mM DTNB with 50 mM NaOH for 15 min at 21 °C; this material was then diluted 1:50 in PBS buffer. Twenty µL of the chloramine samples were then pipetted into a 96-well plate, and the reaction was initiated by adding 180 µL of the TNB solution and incubating for 10 min in the dark at 21 °C. The loss of TNB was quantified by its absorbance at 412 nm using ε 14,150 M^−1^ cm^−1^ [42]. The reactions of chloramines with proMMP9 were carried out by mixing proMMP9 (13 nM) in Buffer A with 20 µL of the chloramine solutions and incubating for 24 h at 37 °C, before the assay of enzyme activity, as described above.

### 2.5. Enzymatic Generation of HOCl by a Myeloperoxidase System

HOCl was also generated using an MPO/H_2_O_2_/Cl^−^ system consisting of 20 nM MPO, 150 mM Cl^−^ and 20 µM H_2_O_2_, with the reactions carried out at a pH of 7.0 and 21 °C in 5 mM phosphate buffer for 2 h. The yield of HOCl formed was quantified by scavenging the HOCl formed using 10 mM taurine and measuring the concentration of taurine chloramines using the TNB assay, as described above [43].

### 2.6. Statistical Analyses

Data are presented as mean values ± SDs from at least three independent experiments. Gel images are representative examples from three experiments. Statistical analyses were performed using GraphPad Prism (version 9.0, GraphPad Software, San Diego, CA, USA) using a one-way ANOVA with Tukey’s multiple comparisons test or Dunnett’s multiple comparisons test, as indicated in the figure legends. Statistical significance was assumed at the *p* ˂ 0.05 level, and it is indicated by * or #, as noted in the legends.

## 3. Results

### 3.1. Activation of proMMP9 by HOCl

**Figure 1:**
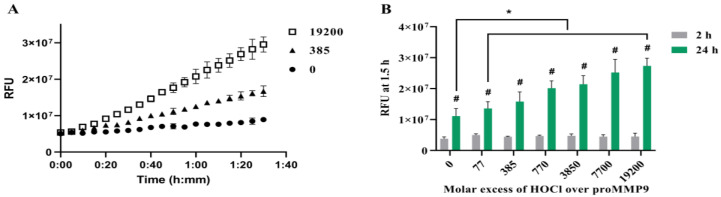
Activation of recombinant human proMMP9 by HOCl. (**A**) Plot of relative fluorescence units (RFUs) against time for reaction of 13 nM proMMP9 exposed to 0, 385- and 19,200-fold molar excesses of reagent HOCl (as indicated, corresponding to 0, 5 µM and 250 µM HOCl, respectively) in Assay Buffer A (containing HEPES) for 24 h at 37 °C before addition of the substrate and recording of the fluorescence intensity of the cleaved peptide over a period of 90 min at 5 min intervals. (**B**) RFUs detected on incubation of proMMP9 with the indicated molar excesses of HOCl for 2 h (grey bars) or 24 h (green bars) at 37 °C before addition of the profluorescent substrate and analysis of fluorescence from the cleaved peptides (cf. Panel A, and Section 2). Data are presented as mean values ± SDs from three independent experiments. Statistical analysis in Panel B was performed using two-way ANOVA with Šídák’s multiple comparisons test (#), and two-way ANOVA with Dunnett’s multiple comparisons test (*). * Indicates statistical significance against the nontreated control sample after 24 h incubation. # Indicates statistically significant differences (*p* < 0.05) between the 24 h- and 2 h-treated samples at the same molar excess of HOCl.

To determine whether reagent HOCl can activate recombinant human proMMP9, samples of the proenzyme were incubated with HOCl at varying molar excesses for either 2 h or 24 h, before the addition of the profluorescent peptide probe and the subsequent determination of the fluorescence from the cleaved peptide over a 1.5 h period (Figure 1).

The initial experiments examined the potential interference from the presence of HEPES in the sample buffer (Buffer A) that is commonly used in experiments that examine MMP activity (see, e.g., [23]), as this compound can react with HOCl [44], although with a rate constant ~10^5^-fold less rapidly than with free Cys residues [45]. The differences in these values are consistent with the preferential targeting of the protein. The activation of proMMP9 by the mercury-derived activating agent APMA (*p*-aminophenylmercuric acetate) was also examined in both Buffer A and the borate buffer (pH 7.8) containing 150 mM of NaCl and 5 mM of CaCl_2_ (as present in Buffer A). These experiments (see Supplementary Figure S1) showed no statistical difference in the time course or extent of APMA-mediated MMP activation, indicating that both buffers supported MMP activity equally.

Subsequent experiments examined the activation of proMMP9 by HOCl at various molar excesses over the concentration of proMMP9 by reagent HOCl in both borate and HEPES-containing buffers (see Section 2). Both reaction systems showed that the relatively modest excesses of HOCl over the protein concentration induced an increase in MMP activity (Figure 1 and Supplementary Figure S2), although in the case of the borate-buffer system, a decrease in the extent of activation was detected with high oxidant excesses, which was less marked in the case of the HEPES buffer. This may be due to the slightly different pH values of these two buffers (7.8 versus 7.4). The stimulation of MMP activity was significantly greater at 24 h when compared with the 2 h treatment (Figure 1).

A potential role for HEPES-derived species (e.g., chloramines) is the observed behavior that was investigated by generating such species by the pretreatment of the HEPES buffer with HOCl at a molar ratio of 5:1 for 15 min, assaying for the resulting chloramines (see Section 2), and then incubating the HEPES chloramines with proMMP9 at a wide range of molar excesses for 24 h, before assaying for enzymatic activity. Under these conditions, no significant activation of proMMP9 was detected (see Supplementary Figure S3, cf. data reported below for other chloramines). Together, these data suggest that HEPES buffer and HEPES-derived species (possibly chloramines) do not significantly modulate the activation of proMMP9 by HOCl. In light of these data, further experiments employed the well-established HEPES-buffer system (Buffer A), as this allowed the experiments to be carried out at a pH of 7.4.

The incubation of the proMMP9 for increasing time periods in the absence of added oxidant in Buffer A resulted in a slow and limited cleavage of the profluorescent peptide substrate (Figure 1A, black circles); this is ascribed to the slow autoactivation of the protein. In contrast, treatment with increasing concentrations of HOCl enhanced the substrate cleavage (Figure 1A,B). This effect was not observed when the profluorescent peptide was treated with HOCl in the absence of proMMP9 (data not shown). After 24 h of pretreatment with HOCl, a greater extent of proMMP9 activation was detected when compared with the pretreatment for 2 h (green bars versus grey bars, Figure 1B). For the 2 h oxidant treatment, an elevated level of activity was detected with the lowest excess of HOCl examined (77-fold, 1 µM), but this did not increase with higher concentrations, which is unlike the situation with the 24 h oxidant treatment, where a clear concentration dependence was detected. These data suggest that the full activation of proMMP9 requires a time period > 2 h under the conditions employed.

At higher HOCl excesses (data not shown), no further increase in activity was detected over that shown in Figure 1B, and, in contrast, the activity decreased slowly (with increasing oxidant concentrations) to the control levels. Together, these data indicate that HOCl can activate proMMP9 at modest HOCl concentrations, but that the active enzyme is susceptible to inactivation at very high HOCl concentrations.

**Figure 2:**
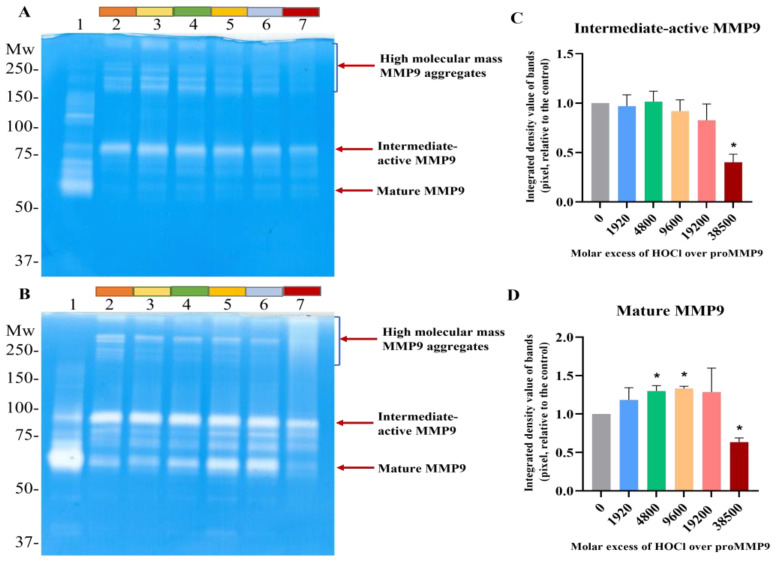
Effect of HOCl on proMMP9 activation detected by gel zymography. (**A**,**B**) 52 nM proMMP9 was exposed to either 1 mM APMA (positive control: Panels A and B, Lane 1) or increasing molar excesses of reagent HOCl (0, 1920, 4800, 9600, 19,200 and 38,500; Lanes 2–7) for 2 h (Panel A) or 24 h (Panel B) at 37 °C. The control and oxidized samples were then subjected to gel zymography, as described in the Section 2. The absence of blue staining (i.e., white bands) indicates the presence of active MMP9 isoforms, as this indicates the degradation of the gelatin in the gel and, therefore, a decrease in protein staining. (**C**,**D**) Image analysis of the band densities from the 24 h activation zymogram gel shown in Panel B. Band intensities of an active intermediate form of MMP9 (see Panel B) are shown in Panel C, and for the mature-form MMP9 in Panel D. Data are presented as means ± SDs from three independent experiments, and gel images are representative examples. Statistical analysis was performed using one-way ANOVA with Dunnett’s multiple comparisons test, with significance against the nontreated group at the *p* < 0.05 level indicated by *. The positions of the molecular-mass markers are indicated on the left of each gel image.

Gel zymography was carried out to both corroborate the fluorescence-assay data, and to examine the isoforms of active MMP9 present in the samples. The treatment of proMMP9 with APMA was used as a positive control (Lane 1 in Figure 2A,B). This treatment resulted in enzyme activation, with the extent of activation (as judged by the intensity of the white bands on the gels) being greater with 24 h pretreatment than 2 h, and with a greater conversion of the proform to the final mature isoform (band at ~67 kD), rather than the active intermediate form (~92 kD, believed to be the proform with an oxidized Cys residue on the propeptide) or other active forms at intermediate molecular masses.

The samples pretreated with HOCl for 2 h (Figure 2A) or 24 h (Figure 2B) showed the significant formation of the active intermediate species (band at ~67 kD). A low level of the active intermediate form was also detected in the nonoxidant-treated samples, probably due to the extended incubation time, and the conditions used to prepare the samples for gel electrophoresis. Significantly greater band intensities were detected for all samples after 24 h incubation when compared with 2 h (Figure 2B), suggesting that the extended time period results in greater autocleavage and activation. No significant increase in the band intensity of the active intermediate form was detected with increasing HOCl concentrations (Figure 2C), possibly because of the subsequent conversion to other forms. Thus, pretreatment with increasing concentrations of HOCl resulted in an increased extent of the conversion of the proMMP9 to the fully active mature form, as indicated in Figure 2D. At the highest excesses of HOCl, a significant decrease in the band intensity of mature MMP9 was detected, consistent with the loss of activity detected in the fluorescence-assay experiments (see above). Interestingly, high-molecular-mass aggregates with enzyme activity (i.e., white bands) were increasingly detected at high HOCl concentrations (Figure 2D).

### 3.2. Inactivation of Active MMP9 by HOCl

**Figure 3:**
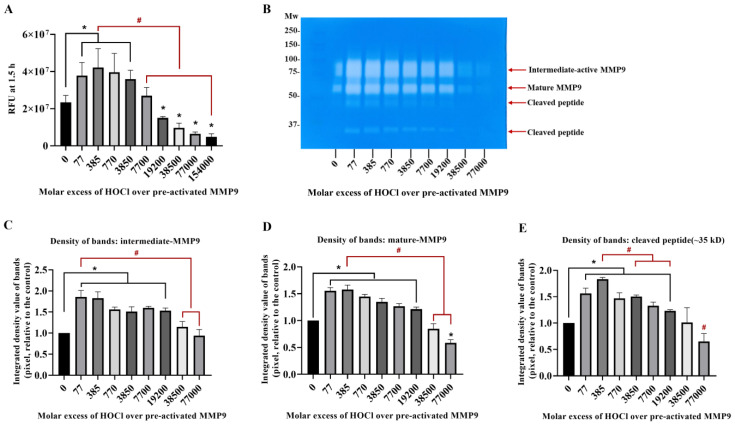
Fluorescence and zymography analyses of the effects of HOCl on preactivated MMP9. Commercial active MMP9 (13 nM) was mixed with increasing molar ratios of reagent HOCl (as indicated) in assay buffer for 24 h at 37 °C before assessment of activity using the fluorescence assay (Panel (**A**)), as described in Section 2 and the legend to Figure 1, although with the activity assessed over 30 min at 1 min intervals. The activity of the samples was also examined using gel zymography (Panel (**B**); see Section 2). Panels C–E present the integrated image densities of the bands corresponding to the gels presented in Panel B, for the bands corresponding to active intermediate MMP9 (Panel (**C**)), mature-form MMP9 (Panel (**D**)) and cleaved peptide (Panel (**E**)), assessed using ImageJ software. Quantitative data are presented as means ± SDs from three independent experiments. Statistical analysis was performed using one-way ANOVA with Dunnett’s multiple comparisons test, with statistical significance (assumed at the *p* < 0.05 level) against the untreated group (0 µM) indicated by *, and against the 77- or 385-fold molar excesses of HOCl (the highest band intensities detected) indicated by #.

To confirm that active MMP9 is inactivated by HOCl at high concentrations, commercial active recombinant human MMP9 was treated with increasing concentrations of reagent HOCl, in a similar manner to the above-described experiments, using the fluorescence-activity assay with a 24 h treatment. Interestingly, the treatment of this (supposed) activated enzyme with an increasing HOCl molar excess over the range of 77–3850 fold (1–50 µM) resulted in an increase in the MMP activity, indicating that the commercial sample contained significant levels of suboptimally activated MMP9 (Figure 3). However, higher concentrations of HOCl decreased the MMP activity to the control or lower levels (Figure 3A), confirming that high concentrations of HOCl inactivated MMP9 over the 24 h incubation period examined.

Corresponding zymography experiments (Figure 3B) confirmed that the exposure of the active MMP9 to increasing HOCl concentrations resulted in the further activation and cleavage of the MMP9. Thus, a greater extent of the conversion of the proform to the intermediate-active (Panels B and C; band at ~92 kD) and mature forms (Panels B and D; band at ~67 kD), and an increased extent of the formation of cleaved but still active forms of the protein at ~47 and ~35 kD (Panels B and E) were detected.

### 3.3. Effect of TIMP1 on MMP9 Activity

**Figure 4:**
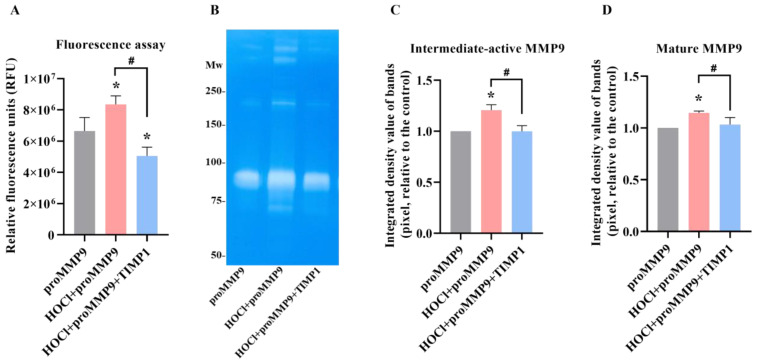
Fluorescence activity and zymographic detection of proMMP9 exposed to HOCl and, subsequently, TIMP1. (**A**) ProMMP9 (13 nM) was incubated with a 385-fold molar excess of reagent HOCl (5 µM) for 2 h at 37 °C, at pH 7.4, in buffer, and then incubated with a 10-fold molar excess of TIMP1 (over proMMP9) for a further 1 h before the assay of activity using either the fluorescence-activity assay (see legends to Figure 1 and Figure 3 for further details) or (**B**) zymography. The data in Panel A are presented as relative fluorescence units and are the means ± SDs of three technical replicates from three biological replicates. (**C**,**D**) Integrated density value of the bands from Panel B were analyzed using ImageJ software and are expressed as means ± SDs from three independent experiments. Statistical analysis was performed using one-way ANOVA with Dunnett’s multiple comparisons test. Statistical significance at the *p* < 0.05 level when compared to the nontreated group is indicated by *. Statistical significance of the TIMP1-treated samples compared with the HOCl-treated proMMP9 is indicated by #.

TIMP1 is a member of the family of endogenous glycoproteins (TIMP1-3) that act as endogenous inhibitors of MMPs. Consequently, it was of interest to determine whether TIMP1 might modulate the activity of HOCl-activated MMP9. ProMMP9 was exposed to a 385-fold molar excess of HOCl for 2 h to activate the enzyme (cf. data presented above), with the activated species then incubated with a 10-fold molar excess (over the concentration of proMMP9) of TIMP1 or buffer before the assay using the profluorescent substrate. As shown in Figure 4A, the treatment with HOCl activated the enzyme in a significant manner, and the subsequent incubation with TIMP1 diminished the enzymatic activity to the control levels or lower (consistent with limited autoactivation (see above)).

This effect was corroborated by zymography, where the enhanced gelatin cleavage induced by the HOCl-mediated activation of proMMP9 was inhibited by a 10-fold molar excess of TIMP1 (Figure 4C). TIMP1 also inhibited the activity of the aggregated species. These data confirm that both the fluorescence and zymography activities are enzymatic in nature and inhibited by classical MMP9 inhibitors.

### 3.4. Activation of proMMP9 by MPO/H_2_O_2_/Cl^−^

These studies were subsequently extended to determine whether the HOCl generated by the complete enzyme system was able to activate proMMP9 in a similar manner to the reagent oxidant. The concentrations of HOCl formed were modulated by altering the concentration of H_2_O_2_ added into the reaction system over the range of 1–20 µM. The yield of HOCl formed under these conditions was determined by the addition of taurine (in the absence of proMMP9), which reacts with HOCl to give long-lived taurine chloramine, with this quantified using TNB. Thus, taurine (10 mM) was incubated with the MPO system (20 nM MPO, 150 mM NaCl, 20 µM H_2_O_2_ in 5 mM phosphate buffer (pH 7.0)) for 2 h at 37 °C. Under these conditions, the yield of taurine chloramine, based on the H_2_O_2_ supplied, is essentially quantitative (i.e., the yield of HOCl was ~20 µM) [46].

**Figure 5:**
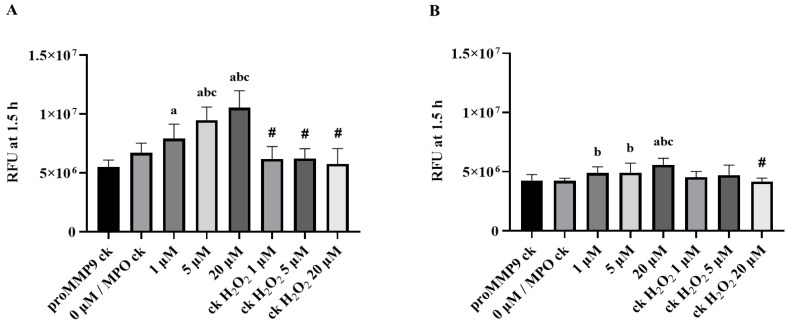
Effect of an MPO/H_2_O_2_/Cl^−^ enzymatic-reaction system on the activation of proMMP9, as detected by cleavage of a profluorescent substrate by active MMP9. (**A**,**B**) An amount of 13 nM proMMP9 was mixed with an enzymatic MPO system (20 nM MPO; 1, 5 and 20 µM H_2_O_2_) in Assay Buffer A for 2 h (**A**) or 24 h (**B**) at 37 °C, before assessment of MMP9 activity, over a 1.5 h period, in the presence of 20 µM substrate, with measurements taken every 5 min with λ_ex_ 320 nm and λ_em_ 405 nm. Data are presented as RFUs and are means ± SDs of three technical replicates from each of three biological replicates. Statistical analysis was performed using one-way ANOVA with Tukey’s multiple comparisons test. Statistical significance compared to proMMP9 (bars labeled rhMMP9 ck) alone, proMMP9 incubated with MPO alone (bars labeled 0 μM/MPO ck) and the full enzyme system with 1 µM and 5 µM H_2_O_2_ are indicated by a, b, and c, respectively. Statistically significant differences between the H_2_O_2_-alone groups compared with the corresponding complete enzyme system-treated groups are indicated by #.

Based on these data, the effect of the MPO system on proMMP9 was examined after incubation for 2 or 24 h at 37 °C (Figure 5). The extent of the substrate cleavage by active MMP9 was compared with the control groups containing proMMP9 and MPO (0 µM H_2_O_2_), various concentrations of H_2_O_2_ with proMMP9 (no MPO) and proMMP9 alone (no MPO or H_2_O_2_). The resulting data indicate that the intact enzyme system with 1–20 µM H_2_O_2_ significantly increased the MMP9 activity after both the 2 and 24 h activations, and in a H_2_O_2_-concentration-dependent manner. This activation was much more extensive at the 2 h time point than at 24 h (unlike the HOCl system). The lower level of activity at 24 h is consistent with a subsequent inactivation of the active MMP activity by the oxidants generated by the enzyme. In contrast to the complete system, treatment with MPO alone, H_2_O_2_ alone or the extended incubation period did not stimulate MMP9 activity under these conditions.

### 3.5. Effect of Methionine on Activation of proMMP9 by HOCl

To further examine the role of HOCl in activating proMMP9, experiments were carried out in the presence of methionine (Met) (1 mM), which reacts with HOCl with a high-rate constant (*k* ~3 × 10^7^ M^−1^ s^−1^ [45]) to give the corresponding (unreactive) sulfoxide. Met was added into the proMMP9 mixture either before the addition of reagent HOCl or after the 2 h reaction.

**Figure 6:**
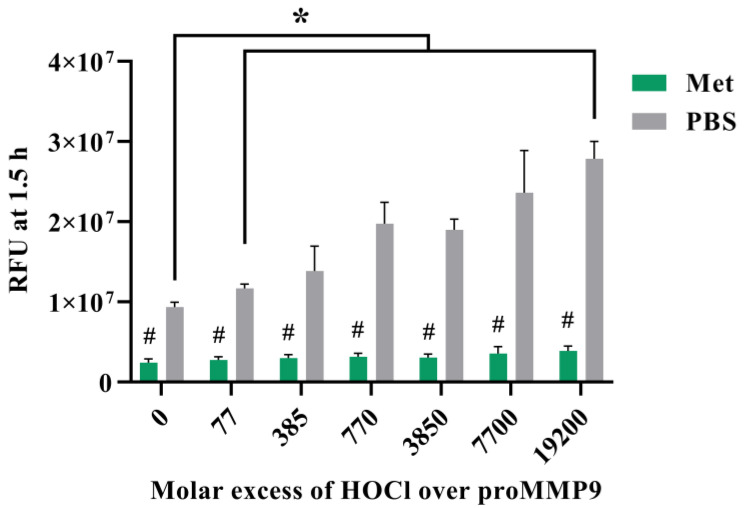
Effect of methionine on HOCl-mediated activation of proMMP9. Methionine (1 mM, or PBS for the control samples) was mixed with 13 nM (1 μg mL^−1^) proMMP9 before exposure to increasing molar ratios of reagent HOCl over protein (as indicated) in Assay Buffer A for 24 h at 37 °C. The activity of the MMP9 samples was then assayed as described earlier. The data are presented as RFUs, and are the means ± SDs of three technical replicates from each of the three biological replicates. Statistical analysis was performed using two-way ANOVA with Dunnett’s multiple comparisons test and Šídák’s multiple comparisons test. Statistical significance compared with the untreated group (0 µM HOCl) is indicated by *, and # indicates statistical significance between the Met and PBS treatments.

The data obtained (Figure 6) show that, under these conditions, Met is effective at preventing the activation of the proMMP9 when compared with the control condition (addition of PBS in place of Met) when added before the oxidant. Smaller extents of inhibition were detected when the Met was added after 2 h (Supplementary Figure S4). Significant differences between the samples with added Met (at time zero) and controls were detected at all the concentrations of HOCl employed. The presence of Met was not completely efficient at preventing activation, with low extents of proMMP9 activation still detected, suggesting that proMMP9 competes with Met, to a small extent, for the added HOCl, and is consistent with the very rapid reaction of HOCl with proMMP9.

**Figure 7:**
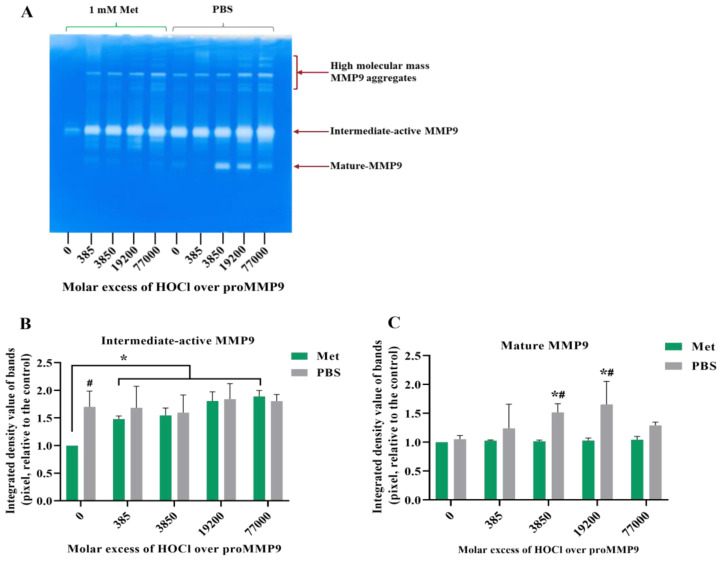
Effect of Met on the activation of proMMP9 by HOCl as detected by zymography. (**A**) proMMP9 (52 nM, 4 μg mL^−1^) was mixed with 1 mM Met or PBS (control) before the addition of the indicated molar excesses of reagent HOCl over protein and subsequent incubation for 24 h at 37 °C, before analysis of the MMP9 activity by gel zymography (see Section 2) with 20 ng proMMP9 loaded per lane and the development of the gel overnight at 21 °C. A representative gel from three independent experiments is presented. (**B**,**C**) Densitometric data from the combined dataset for the bands at ~92 kD, assigned to the active intermediate form of MMP9 (Panel B), and the band at ~67 kD assigned to mature MMP9 (Panel C), as determined using ImageJ software. Data are mean values ± SDs from three independent gels. Statistical analysis was performed using two-way ANOVA with Dunnett’s multiple comparisons test and Šídák’s multiple comparisons test. Statistical significance compared to the untreated group (0 µM HOCl) is indicated by *; # indicates statistical differences between the Met- and PBS-containing samples.

Gel zymography was used to confirm the activation of proMMP9 by HOCl, and the effects of Met (Figure 7). The exposure of proMMP9 to increasing molar excesses of HOCl, in the absence of Met, resulted in a significant loss of gelatin staining at various regions of the zymography gels, which is consistent with the presence of activated enzyme. In particular, a loss of staining was observed at ~92 kDa and ~67 kDa, consistent with the presence of an active intermediate form and mature MMP9, respectively. Multiple additional (white) bands were detected near the top of the gel, indicating the presence of aggregates with enzymatic activity. The presence of 1 mM of Met (added before HOCl) did not markedly inhibit the formation of the 92 kDa form, but did inhibit the formation of mature MMP9 (at ~67 kDa; Figure 7A,C). The presence of Met also diminished the quantity of active aggregates at the top of the zymography gels (Figure 7A). These data are consistent with the rapid reaction of HOCl with proMMP and the incomplete scavenging of HOCl by Met.

### 3.6. Effect of Amino Acid-Derived Chloramines on proMMP9

Although the above data indicate that the reaction of proMMP9 with HOCl is rapid, in biological systems, there is an abundance of alternative targets and, hence, the flux of HOCl to which proMMP9 might be exposed may be limited by competition with other materials. Reactions with other proteins (as well as free amino acids and peptides) is likely to be significant due to their high abundance [47], with these reactions likely to give chloramines (RNHCl species) from abundant Lys, His and Arg residues. Many chloramines retain the oxidizing capacity of HOCl, and therefore it was of interest to determine whether these species, which vary in reactivity, activate proMMP9 (cf. data for HEPES chloramines presented in Supplementary Figure S3).

**Figure 8:**
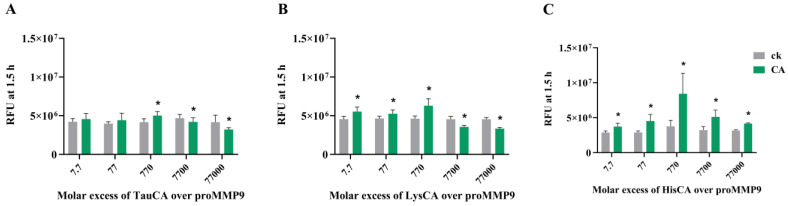
Effects of different concentrations of amino acid chloramines (green bars) formed on taurine (TauCA) (Panel (**A**)), lysine (LysCA) (Panel (**B**)) and histidine (HisCA) (Panel (**C**)), and parent amino acids (gray bars labeled ck), on proMMP9 activation, as determined by cleavage of a profluorescent peptide and the determination of the consequent fluorescence. Preformed chloramines were generated by adding HOCl to a 5-fold excess of taurine and *N*α-acetyl-lysine, or a 2-fold excess of *N*α-acetyl-His-NHMe, in 5 mM sodium phosphate buffer (pH 7.0). Chloramine yields were then quantified using TNB assay (see Section 2), and the indicated concentrations of chloramines were then incubated with proMMP9 (13 nM) for 24 h at 37 °C before the quantification of the enzyme activity by fluorescence spectroscopy. Data are presented as the mean RFU values ± SDs from three technical replicates from each of the three biological replicates for the chloramine-containing samples versus the corresponding parent amino acid-treated controls (labeled “ck”). Statistical analysis was performed using unpaired *t* tests with Welch’s correction, with the statistical significance between the paired samples indicated by *.

These data indicate that preformed chloramines on taurine (TauCA), *N*α-acetyl-lysine (LysCA) and *N*α-acetyl-His-NHMe (HisCA) can activate proMMP9 (Figure 8), unlike HEPES chloramines (see above). In each case, activation was observed at modest concentrations of these species, followed by a plateau and a subsequent decrease in activity, consistent with both activations, and subsequently the deactivation of active MMP9 once formed. The greatest extent of activation was detected with HisCA, and the lowest with TauCA.

### 3.7. Effect of Human Serum Albumin-Derived Chloramines on proMMP9

**Figure 9:**
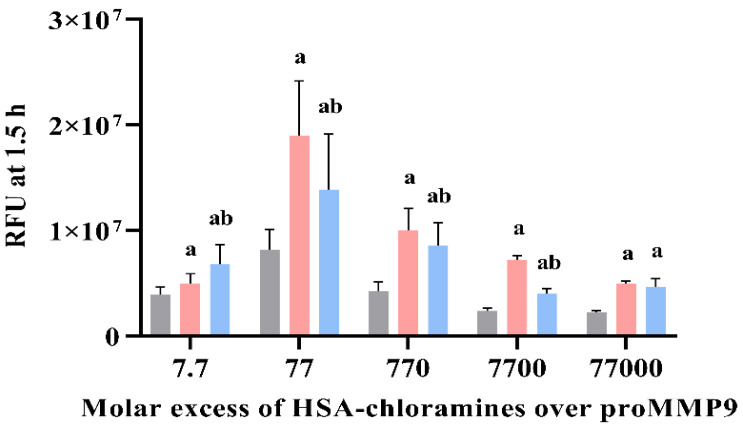
Effect of HSA chloramines on the activation of proMMP9 as determined by fluorescence assay. Chloramines were generated on HSA (40 µM) exposed to a 50-fold molar excess of HOCl (2 mM), with chloramine formation and concentration determined using the TNB assay. Defined molar excesses of the preformed chloramines (as indicated; pink bars), or chloramine solutions treated with 1 mM Met for 5 min (blue bars), or comparable concentrations of parent protein (gray bars) were then incubated with proMMP9 (13 nM) for 24 h at 37 °C before the determination of the extent of the cleavage of the profluorescent peptide substrate by fluorescence spectroscopy. Data are presented as RFUs and are means ± SDs of three technical replicates from three biological replicates. Statistical analysis was performed using one-way ANOVA with Tukey’s multiple comparisons test. Statistical significance compared with the HSA control is indicated by a, and differences relative to the chloramine-treated samples are indicated by b.

To determine whether proMMP9 activation also occurs with protein-derived chloramines, similar studies were carried out with human serum albumin (HSA) treated with a 50-fold molar ratio of HOCl over HSA (or PBS for controls), as described previously [41]. The chloramine formation and their concentration were assessed using TNB (see Section 2). ProMMP9 was treated with defined concentrations of HSA chloramines or comparable concentrations of PBS-treated HSA, over the concentration range 100 nM–1 mM (Figure 9). Additional experiments were also carried out with chloramine samples pretreated with 1 mM of Met before the addition to proMMP9 (blue bars, Figure 9). The enzyme activation was subsequently assessed by fluorescence.

The data obtained (Figure 9) show that HSA chloramines can activate proMMP9, with the significant stimulation of the substrate cleavage detected with all the HSA chloramine concentrations tested, when compared with the controls containing only the parent protein at an identical concentration. In all cases, with the exception of the highest chloramine concentrations (1 mM), this stimulation was abrogated by Met (1 mM) added to the chloramine samples before the addition to the proMMP9. These data are consistent with the activation of proMMP9 by chloramines, and not downstream products. The low level of protection afforded by Met with the highest HSA chloramine concentrations is not surprising, as the chloramine and Met concentrations were equimolar, and so incomplete chloramine removal is likely. These data indicate that HSA-derived chloramines are effective activators of proMMP9 and have more marked effects than amino acid chloramines (Figure 8 vs. Figure 9).

### 3.8. Effects of Extracellular Matrix and Matrix-Protein-Derived Chloramines on proMMP9

In light of the above data, studies were undertaken with chloramines generated on two proteins that are abundant in ECM matrices (fibronectin (FN) and laminins), as well as the mixture of proteins present in basement membrane ECM (“basement membrane extract” (bme)). Both FN and laminin are known targets of MPO-derived oxidants, both in vitro and in vivo (e.g., in advanced human atherosclerotic lesions [5,11,14,46,48]), and hence, would be expected to be major sites of chloramine formation.

**Figure 10:**
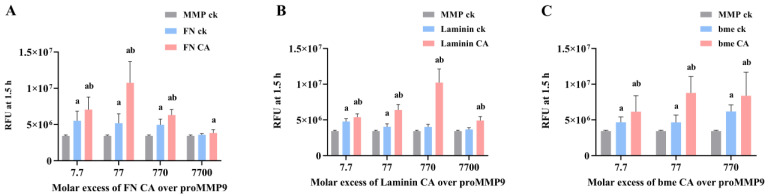
Effects of chloramines present on purified fibronectin, laminin-111 and mixture of ECM proteins present in the murine basement membrane extract (bme) on proMMP9 activation detected by fluorescence assay. Chloramines were generated by adding a 50-fold molar excess of HOCl to: (**A**) human plasma fibronectin (FN), (**B**) murine laminin-111 and (**C**) murine bme. Chloramine formation and concentrations were determined using the TNB assay, as outlined in Section 2. The preformed chloramines (at the indicated molar excesses over the proMMP9 concentration; pink bars labeled CA), or comparable concentrations of the parent proteins (blue bars, labeled ck), were then incubated with proMMP9 (13 nM) for 24 h at 37 °C, before the determination of the extent of the cleavage of the added profluorescent substrate by fluorescence detection. Controls with no added protein or chloramines (gray bars, labeled MMP ck) were also examined. Data are presented as means of the RFUs ± SDs of three technical replicates from each of the three biological replicates. Statistical analysis was performed using one-way ANOVA with Tukey’s multiple comparisons test. Statistical significance compared with the MMP control is indicated by a, and b indicates statistical differences compared with the native protein controls.

The data obtained (Figure 10) indicate that chloramines present on FN (FNCA) on murine laminin-111 (laminin CA) and on ECM proteins in the murine bme (bme CA) can activate proMMP9. This occurred with all the chloramine concentrations tested (100 nM–100 µM), except with the highest concentration of FN chloramines. In each case, the enzymatic activity was greater than that observed for proMMP9 incubated in the absence of added proteins, or proMMP9 incubated with native proteins. Interestingly, the native proteins also induced small increases in the proMMP9 activity, possibly due to the presence of contaminating proteases in the commercial preparations. The extent of proMMP9 activation varied between the protein chloramines, with FN chloramines showing maximum stimulation at a 77-fold molar excess (i.e., 1 µM chloramine), and laminin chloramines at a 770-fold molar excess (10 µM chloramines). No clear maximum was detected with the bme chloramines. These differences may reflect the type (i.e., whether these are formed on Lys, His, Arg or the N-terminal amines) and location (i.e., sequence position) of the chloramines on these proteins. With both the FN and laminin chloramines, a significant decrease in the MMP activity was detected with the highest chloramine concentrations, suggesting that these species can both activate and subsequently deactivate the enzyme.

**Figure 11:**
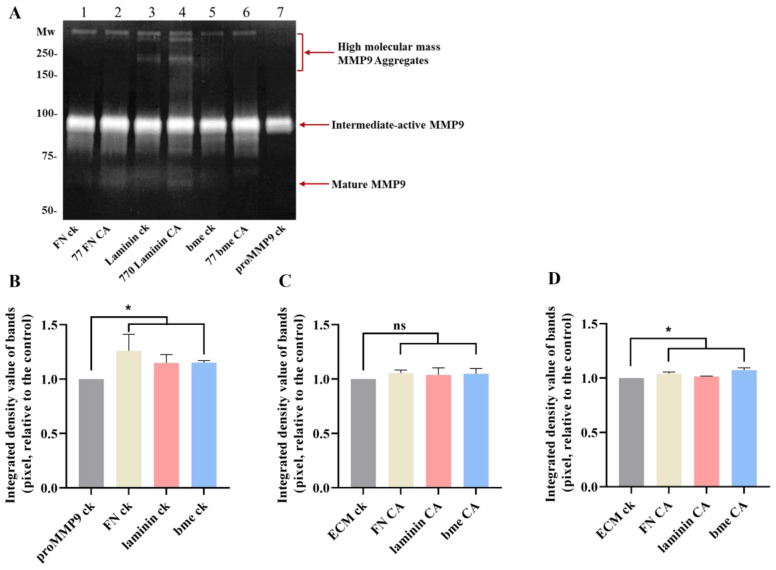
Effect of ECM protein chloramines on proMMP9 activation determined by gel zymography. Chloramines were generated on FN, laminin and bme, as described in the legend to Figure 10, and we then selected molar excesses of these chloramines (77-fold molar excess for FN CA and bme CA, 770-fold molar excess for laminin CA, as indicated), or comparable concentrations of the parent proteins (indicated as FN ck, laminin ck and bme ck), incubated with proMMP9 (26 nM, 2 μg mL^−1^) for 24 h at 37 °C. Samples of proMMP9 were also incubated in the absence of added protein or chloramines (lane labeled proMMP9 ck). The chloramine concentrations chosen for this study are those which showed the maximum activity (see Figure 10). After incubation, the samples were subjected to: (**A**) Gel zymography (see Section 2), with a representative gel image presented from 3 independent experiments. Panels B–D present densitometry data from the combined dataset for: (**B**) the active intermediate MMP9 band of FN, laminin and bme control at ~92 kD compared with proMMP9 ck; (**C**) the active intermediate MMP9 band of FN CA, laminin CA and bme CA at ~92 kD, compared with the corresponding nontreated protein controls; (**D**) the mature MMP9 band at ~67 kD, as determined by ImageJ analysis. The densitometric data are mean pixel intensities ± SDs expressed relative to the control (lane labeled proMMP9 ck) from 3 independent experiments. Statistical analysis was performed using unpaired *t* tests with Welch’s correction for multiple comparisons, with statistical significance compared with the control indicated by *.

The effects of ECM protein chloramines on proMMP9 were further examined by gel zymography, using the chloramine concentrations determined to give the highest extent of proMMP9 activation (cf. Figure 10). These experiments confirmed that proMMP9 was activated to a greater extent by chloramines than the corresponding native proteins (see also Figure 10), or proMMP9 incubated in the absence of any treatment. Thus, increased activity was detected for the active intermediate form (at ~92 kD) and the mature band at ~67 kD, but not for the high-mass aggregates, although weak bands for the aggregated active enzyme were detected in some replicates (Figure 11). Taken together, these fluorescence and zymography-gel data indicate that the chloramines formed on ECM proteins can activate proMMP9, and also, at high concentrations, inactivate the enzyme.

## 4. Discussion

The activation of MMPs (and related ADAM and ADAMTS enzymes of the zinc protease superfamily [49,50]) results in ECM degradation, with the extent of the cleavage of specific components being dependent on the enzyme that is activated, as each has specificity for particular ECM species [51]. Furthermore, different cells express different family members, with MMP9 being expressed at high levels by activated neutrophils. This enzyme is abundant in bone marrow and lymphoid tissues, and at sites of inflammation and wound repair, where neutrophils accumulate and become activated [28,29]. MMP9 (gelatinase B) plays a key role in normal physiology (e.g., in embryonic development, reproduction, cell migration, angiogenesis, neovascularization and bone development), as well as in pathologies, such as arthritis, sepsis, cancer metastasis, cardiovascular diseases and hemorrhage [28,29,38]. MMP9 targets type IV and V collagens, with the former being a major structural and functional component of the basement membranes that underlie all epithelial and endothelial cells [28,29]. MMP9 also plays a key role in the release of the inflammatory cytokine IL-1β [28,29].

The activity of MMP9, and other family members, is tightly controlled in vivo at the levels of secretion, subcellular or extracellular localization, the activation of the zymogen form, the expression of their endogenous protein inhibitors (e.g., TIMPs) and by protease degradation [52]. However, it is becoming increasingly clear that MMP activation can be induced in a nonregulated manner by inflammatory oxidants, such as HOCl [22,23,24], peroxynitrite/peroxynitrous acid [53,54,55], radicals formed by xanthine/xanthine oxidase [24] and the plethora of species formed by activated neutrophils [25,26,27]. This can occur via the “cysteine-switch” mechanism, in which a specific Cys in the propeptide is targeted by the oxidant, resulting in the modification of the thiol group that ligates the active-site zinc atom (to multiple oxidized forms, including sulfenic, sulfininic and sulfonic acids [23]), the release of the prodomain and the exposure of the active site [22,23,24].

**Figure 12:**
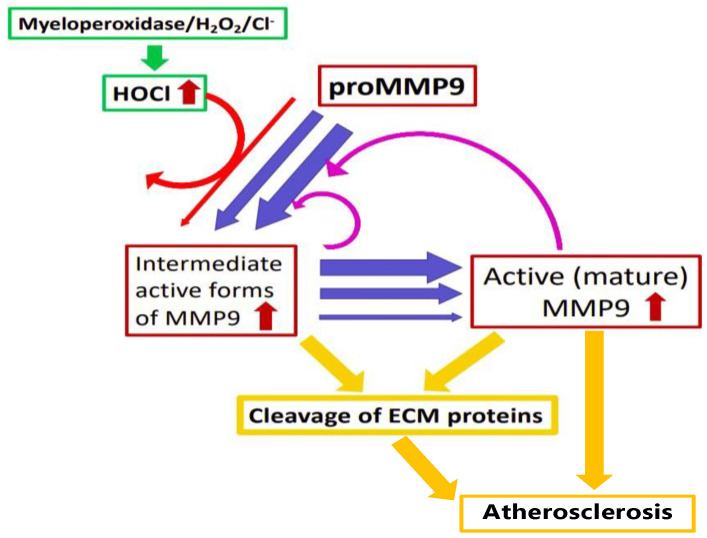
HOCl generated by the myeloperoxidase/H_2_O_2_/Cl^−^ enzyme system, or reagent HOCl, can convert inactive proMMP9 to active intermediate forms of MMP9 (red arrows). These undergo subsequent further processing (possibly involving further oxidation or proteolysis) to active mature MMP9 (blue arrows). The active forms of MMP9 can catalyze the further proteolytic conversion of proMMP9 to active forms (pink arrows), resulting in an increasing cycle of activation of MMP9 (as indicated by the lines of increasing thickness) and the cleavage of target ECM proteins, including collagens IV and V (yellow arrows), exacerbating tissue damage and diseases, such as atherosclerosis.

In this study, both HOCl and an enzymatic MPO/H_2_O_2_/Cl^−^ system were shown to activate proMMP9; this confirms the previous studies carried out with this enzyme [25,26,27]. This activation was inhibited by added Met and TIMP1, which is consistent with the HOCl-mediated effects on enzymatic activity. A greater extent of activation was detected at 24 h compared with 2 h with reagent HOCl, but the converse was seen with the enzymatic system. The increase in activity over time seen with HOCl is consistent with some of the activation that occurs via long-lived species, possibly including chloramines (see below), and also the proteolytic activation of additional proMMP9 by active MMP9 generated by the oxidant-mediated reactions (Figure 12). The extent of activation increased with increasing HOCl concentrations up to a plateau value, after which it decreased. This diminished activity at very high HOCl concentrations is consistent with oxidant-mediated enzyme inactivation. The potential inactivation of the active enzyme was tested with commercial active MMP9. The “active” enzyme showed a similar inverted-“U”-shaped response as the proform, though with the highest activity detected at lower oxidant concentrations. This behavior is consistent with the presence of proMMP9 in the commercial preparation of “active” MMP9, with activation followed by inactivation at high oxidant doses. This profile also suggests that proMMP9 activation is a more rapid and efficient process than HOCl-mediated inactivation. The rapidity of the activation process is supported by the diminished effect of Met added 2 h after the initiation of the reaction when compared with Met added at time zero (Supplementary Figure S3). The extent of the protection afforded by the added Met was, however, variable between the different reaction systems, with this likely to reflect the time point at which the Met was added, differences in the chemistry of HOCl and different chloramines, and the role of autoxidation reactions. These data for MMP9 are consistent with previous reports on other MMPs, where activation was detected at low levels of HOCl, and inactivation at high concentrations [23,56]. A comparison of the activation–inactivation curves obtained with HOCl in HEPES buffer versus borate buffer (Supplementary Figure S2) suggests that inactivation is more marked at a given (high) excess of HOCl in borate buffer (e.g., 770 fold or higher) when compared with HEPES. However, as biological systems contain many HOCl-reactive species, the data obtained in HEPES are more likely to reflect the biological reality than completely inert buffers, such as borate.

The extent of the activation of the total pool of proMMP9 present in the reaction mixtures is difficult to accurately assess, as the activity data for the HOCl systems clearly reflect the difference between the activation process and any inactivation. However, a comparison of the difference in the relative fluorescent units (RFUs) for APMA compared with reagent HOCl, carried out on the same batch of proenzymes, indicates that APMA generates about an 8-fold increase in activity, and HOCl generates a ~2–3-fold increase, compared with the untreated enzyme, suggesting that the oxidant system activates 25–30% of the total possible activity (on the assumption that APMA induces 100% activation).

The zymography data support the “cysteine-switch” as the mechanism of activation, as the gels indicate that the initial active species formed from proMMP9 is the so-called active intermediate form (~92 kD), where the Cys-zinc atom ligation has been disrupted, with the mature form of the enzyme (~67 kD) detected at a lower level (as judged by the loss of gelatin staining) and at later time points. Additional active forms were also detected on these gels, including high-molecular-mass aggregates (~200–300 kD) and a further cleaved peptide (at ~40 kD). A potential route to the high-mass aggregates is oxidant-mediated crosslinking (via a disulfide bond) between two Cys residues, previously involved in zinc ligation, on different protein molecules. The nature of these aggregates and the low-intensity cleaved peptide were not examined further. However, it is interesting to note that TIMP1 inhibited both the activity of the monomer species and active aggregates (species of >200 kDa, Figure 4). Inhibition by TIMPs is reported to involve the binding of the TIMP N-terminal domain to the catalytic zinc atom of MMPs [21].

The concentrations of HOCl used here are relatively high when expressed as a molar excess, but in terms of absolute concentrations, these are of potential biological relevance. Thus, the activation of proMMP9 was detected with 1 µM of HOCl (77-fold molar excess), and this increased with HOCl concentrations up to 250 µM. Similarly, the activation was detected with the MPO/H_2_O_2_/Cl^−^ system using MPO supplied with 20 µM of H_2_O_2_ (and hence, a yield of the HOCl of 20 µM). These concentrations are within the range detected within the phagolysosomes of neutrophils (where the HOCl is reported to be mM, although this does not accumulate due to rapid subsequent reactions [57]), and also those detected, or calculated, to be formed at sites of inflammation from extracellular MPO [58]. The high excesses of HOCl (high µM–low mM), where a decrease in the activity of preactivated MMP9 was detected, may be of limited pathophysiological relevance, although such excesses may occur when HOCl is used as a disinfectant. The data obtained also indicate that (commercial) MMP9 preparations can undergo “self-activation” upon extended incubation (cf. differences in the 0 µM bars between 2 and 24 h in Figure 1B and Figure 2), consistent with enzyme instability. This may be due to the presence of low levels of active enzymes in the preparation that (proteolytically) activate more enzymes over time. This interpretation is supported by the gradual increase in the substrate cleavage (Figure 1A) over time for the 0 µM HOCl condition. However, it is clear that reagent HOCl and oxidants (likely HOCl) formed by the MPO/H_2_O_2_/Cl^−^ system also activate proMMP9.

While the above data indicate that HOCl can react directly with proMMP9, possibly via the prodomain Cys residue, to active the enzyme, in complex biological systems, HOCl is also likely to react, in a competitive manner, with other targets. Thus, HOCl reacts with protein side chains, with the order of reactivity being: Cys > Met > cystine > His > α-amino > Trp > Lys > Tyr > Arg > backbone amides [8,45]. In the extracellular environment, Cys residues are low in abundance, and particularly when compared with other reactive residues on proteins (e.g., His, α-amino, Lys and Arg) and free amino acids. These species react to produce chloramines that can retain the oxidizing capacity of HOCl and that target Cys and Met residues [59,60]. Thus, chloramine formation on ECM proteins is likely to be a major and significant process, and particularly as MPO binds avidly to ECM materials, including proteins, glycoproteins, proteoglycans and glycosaminoglycans [3,5,61,62]. The activation of proMMP9 by chloramines may therefore be a significant process in vivo.

The data obtained for chloramines present for free amino acids (Figure 8), HSA (Figure 9), isolated ECM proteins (Figure 10 and Figure 11) and basement membrane extracts (Figure 10 and Figure 11) indicate that these can activate proMMP9 at concentrations as low as 100 nM, although this is dependent on both the chloramine and its concentration, with increasing activation detected with greater concentrations up to a plateau value, after which the activity declined, probably because of MMP9 inactivation. This activation was inhibited by pretreatment with Met, which removes chloramines. The extent of activation mirrors the reactivity and stability of these chloramines, with this decreasing down the sequence: His > Lys > taurine [41,59,60]. Taurine is highly abundant (20–50 mM) in leukocytes [63], and this is released extracellularly (together with MPO) upon the stimulation of neutrophils, resulting in the presence of significant concentrations of both taurine and (presumably) taurine chloramines at sites of inflammation [64]. High concentrations of taurine, and the low reactivity of taurine chloramines, may therefore protect against the effects of HOCl and decrease the extent of proMMP9 activation, although taurine chloramines can also activate the enzyme to a limited extent.

Efficient activation was detected with HSA-derived chloramines, which was greater than with the amino acids, possibly because of favorable interactions between HSA and proMMP9. The nature of the chloramines formed on HSA that induce activation is unknown, with multiple species likely to be present. However, it is well established that HSA is a major plasma target for HOCl [65,66,67], and likely also in other extracellular fluids, with Cys residues targeted preferentially [66]. This may alter the antigen presentation and adaptive immunity [68,69]. The current data indicate that HSA chloramines also target proMMP9, resulting in MMP9 activation.

Efficient proMMP9 activation was also detected with chloramines present on the important and abundant ECM proteins fibronectin and laminin-111, and those formed on basement membrane extracts that contain multiple ECM species. This occurred with chloramine concentrations between 0.1 μM and 100 μM. The data for bme chloramines indicate that even complex three-dimensional ECM assemblies can interact with proMMP9 and give rise to activation. This process may be aided by the association of MMP9 with ECM proteins, given that collagens IV and V are native substrates for this enzyme.

The significance of these observations is increased by the literature data indicating that the HOCl-induced modification of plasma and ECM proteins can induce the cellular expression and release of proMMPs. Thus, the ECM laid down by human coronary artery smooth muscle cells, and subsequently modified by HOCl or an MPO/H_2_O_2_/Cl^−^ system, can upregulate the expression of multiple MMPs in naïve cells (i.e., cells not exposed to the oxidant, only the modified ECM) [14]. HOCl-modified HSA also modulates immune-cell function (reviewed [3,6,70]), with exposure to the modified protein reported to elevate oxidant formation by stimulating neutrophil activation and degranulation at sites of inflammation [71,72]. Other studies have reported that fibronectin fragments (potentially arising from oxidant or enzymatic degradation) and cryptic domains in the α1-chain of laminin-1 exposed by structural changes can induce cellular MMP expression [73,74]. Thus, oxidant formation can result in a feed-forward loop (“vicious cycle”) of increasing MMP expression, release, activation, ECM damage and further activation cycles, with this driven by the synergistic effects of oxidant formation, MMP activation and the further proteolysis of additional proMMP by the oxidant-activated enzyme (Figure 12).

## 5. Conclusions

The data presented here suggest a role for modest concentrations of HOCl (reagent or generated by MPO) and chloramines derived from free amino acids, HSA or ECM species in the conversion of proMMP9 to active MMP9. With high oxidant concentrations, the inactivation of MMP9 can also occur, with this accompanied by structural cleavage and aggregation. The formation of active MMP9 from the proform as a result of oxidation is believed to occur via the oxidation of a Cys residue via the “cysteine-switch” mechanism. This oxidant-mediated activation of proMMP9 may contribute to further proMMP9 expression, release and activation. Thus, the direct inflammation-induced oxidant activation of proMMPs, and the indirect oxidation of proMMPs by ECM and other chloramines, may trigger a complex cycle of tissue damage driven by the synergistic effects of the oxidative activation of the proenzyme, and the subsequent proteolytic activity of active MMP9. The consequences of these processes are likely to be increased ECM degradation and altered cell attachment, proliferation and function. This may exacerbate the capacity of cancer cells to undergo metastasis and result in increased lesion rupture within the artery wall in the context of atherosclerosis and cardiovascular disease.

## Data Availability

All of the data is contained within the article and the Supplementary Materials.

## Supplementary Materials

The following supporting information can be downloaded at: https://www.mdpi.com/article/10.3390/antiox11081616/s1, Figure S1: Activation of recombinant human proMMP9 by APMA in borate buffer vs. HEPES buffer; Figure S2: Comparison of the extent of activation of recombinant human proMMP9 by the indicated molar excesses of HOCl in borate vs. HEPES buffer; Figure S3: Effect of different molar excesses of pre-formed HEPES chloramines on proMMP9 activation as determined by cleavage of a pro-fluorescent peptide and determination of the consequent fluorescence; Figure S4: Effect of methionine added after 2 h on HOCl-mediated activation of proMMP9.
