# Supplementary material for: Activation and Inhibition of Human Matrix Metalloproteinase-9 (MMP9) by HOCl, Myeloperoxidase and Chloramines
Source: Antioxidants (Basel). 2022 Aug 20;11(8):1616. doi: 10.3390/antiox11081616 (PMC9405048; doi:10.3390/antiox11081616)
Supplement: Supplementary file 1 [file antioxidants-11-01616-s001.zip › antioxidants-1857873-supplementary.pdf]

Activation and inhibition of human matrix metalloproteinase-9 (MMP9) by HOCl, myeloperoxidase and chloramines

Yihe Wang [1], Christine Y. Chuang [1], Clare L. Hawkins [1] and Michael J. Davies [1,*]

[1] Department of Biomedical Sciences, Panum Institute, University of Copenhagen, Copenhagen 2200, Denmark

* Correspondence: davies@sund.ku.dk

Supplementary Data

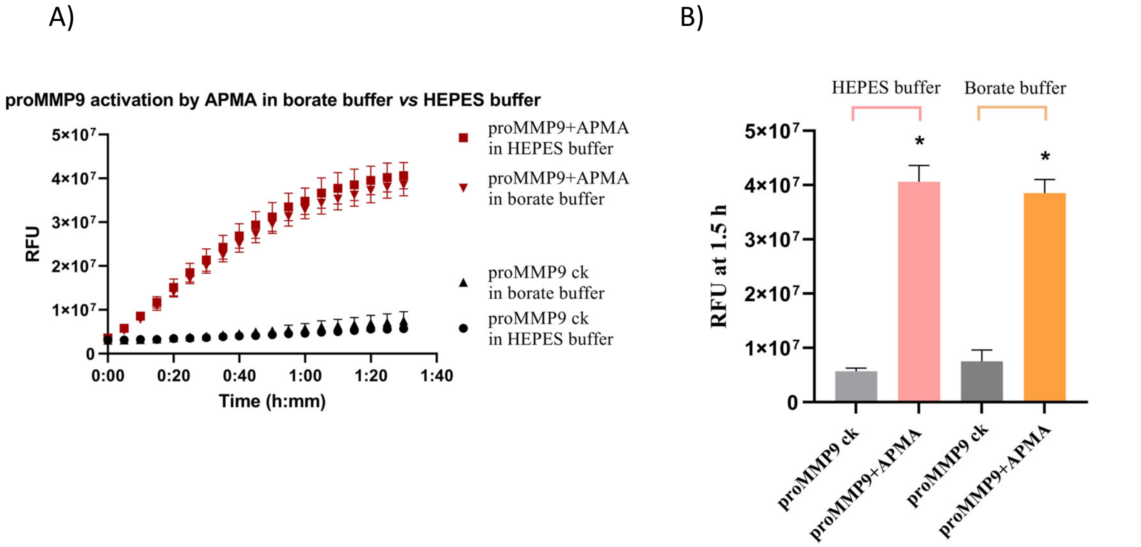

Supplementary Figure S1 Activation of recombinant human proMMP9 by APMA in borate buffer *vs* HEPES buffer. (A) Plot of relative fluorescence units (RFU) against time for reaction of 13 nM proMMP9 exposed to 1 mM APMA in borate buffer and HEPES buffer for 2 h at 37 ºC before addition of the substrate and recording of the fluorescence intensity of the cleaved peptide over a period of 90 min at 5 min intervals. (B) RFU detected on incubation of proMMP9 with the 1 mM APMA for 2 h in HEPES buffer (pink bar) or in borate buffer (orange bar) at 37 ºC before addition of the pro-fluorescent substrate, and analysis of fluorescence from the cleaved peptides (cf. panel A, and Materials and methods). Data are presented as relative fluorescence units (RFU), and presented as mean values ± SD from three independent experiments. Statistical analysis in panel B was performed using one-way ANOVA with Tukey's multiple comparisons test. * Indicates statistical significance against the non-treated control sample after 2 h incubation ($p < 0.05$).

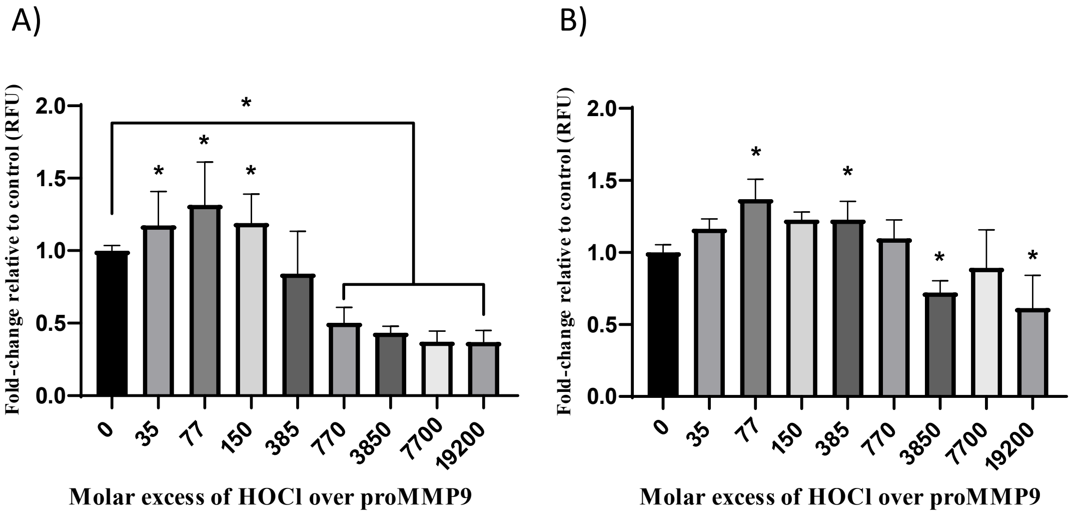

Supplementary Figure S2 Comparison of the extent of activation of recombinant human proMMP9 by the indicated molar excesses of HOCl in borate (left panel) *vs* HEPES buffer (right panel). Data are presented as the fold-change relative to the control samples with no added HOCl (in order to allow easy comparison between the two systems) of the relative fluorescence units (RFU) detected on reaction of 13 nM proMMP9 to the indicated molar excess of HOCl for 24 h at 37 °C before addition of the substrate and recording of the fluorescence intensity of the cleaved peptide over a period of 90 min at 5 min intervals. Data are mean values ± SD from three independent experiments. Statistical analysis was performed using one-way ANOVA with Tukey's multiple comparisons test. * Indicates statistical significance against the control samples with no added HOCl ($p < 0.05$).

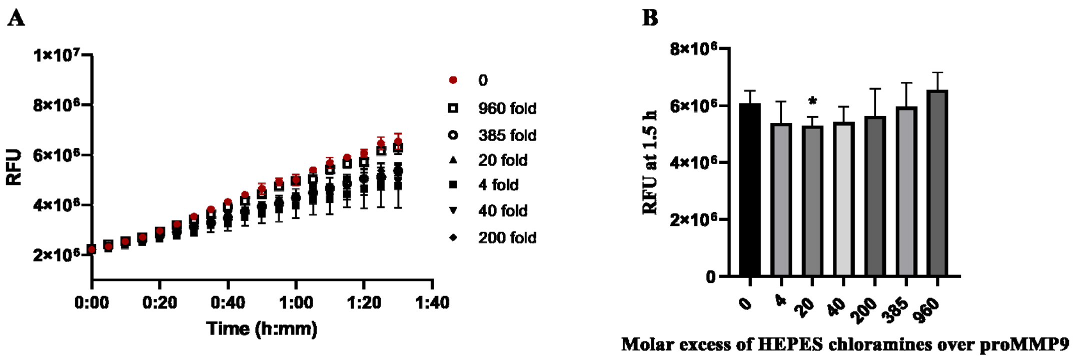

Supplementary Figure S3 Effect of different molar excesses (as indicated) of pre-formed HEPES chloramines on proMMP9 activation as determined by cleavage of a pro-fluorescent peptide and determination of the consequent fluorescence. HEPES chloramines were generated by adding HOCl to a 5-fold excess of HEPES in 5 mM sodium phosphate buffer, pH 7.0, at 4 °C. Chloramine yields were then quantified using TNB assay (see Materials and methods) and the indicated molar excesses of chloramines then incubated with proMMP9 (13 nM) for 24 h at 37 °C before quantification of enzyme activity by fluorescence spectroscopy. Data are presented as the mean RFU values ± SD, from three independent experiments. Statistical analysis was performed using one-way ANOVA with Tukey's multiple comparisons test, with differences at the $p < 0.05$ level indicated by ***.**

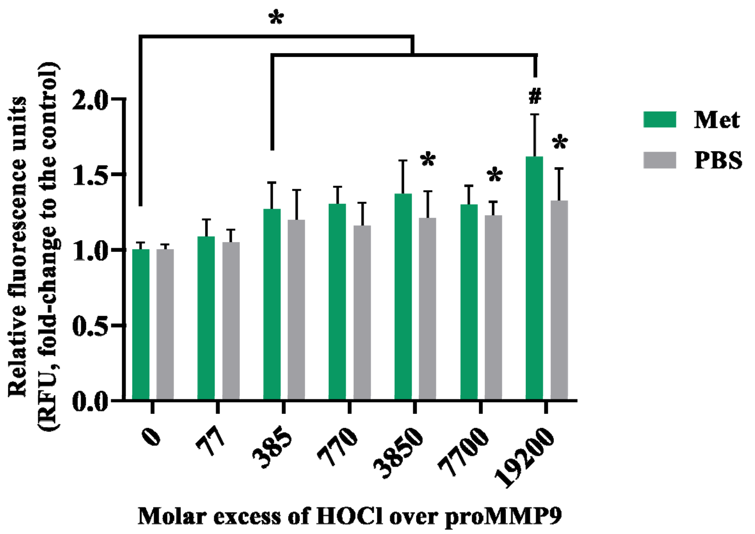

Supplementary Figure S4 Effect of methionine added after 2 h on HOCl-mediated activation of proMMP9. ProMMP9 (13 nM, 1 µg mL^{-1}) was exposed to increasing molar ratios of reagent HOCl over protein (as indicated) in assay buffer A for 2 h then mixed with methionine (1 mM, or PBS for the control samples) and incubated for a further 22 h at 37 °C. The activity of the MMP9 samples was then assayed as described in the Materials and methods. The data are presented as the fold-change relative to the non-treated group (bars labelled 0) and are the mean ± SD of three technical replicates from each of three biological replicates. Statistical analysis was performed using two-way ANOVA with Dunnett's multiple comparisons test and Šídák's multiple comparisons test. Statistical significance compared to the untreated group (0 µM HOCl) is indicated by *, and # indicates statistical significance between the Met and PBS treatments.
